# Oxazin-5-Ones as a Novel Class of Penicillin Binding Protein Inhibitors: Design, Synthesis and Structure Activity Relationship

**DOI:** 10.1371/journal.pone.0163467

**Published:** 2016-10-17

**Authors:** Efeturi Abraham Onoabedje, Akachukwu Ibezim, Sunday Nwankwor Okafor, Ufuoma Shalom Onoabedje, Uchechukwu Chris Okoro

**Affiliations:** 1 Department of Pure & Industrial Chemistry, University of Nigeria, Nsukka, Enugu State, Nigeria; 2 Faculty of Pharmaceutical Sciences, University of Nigeria, Nsukka, Enugu State, Nigeria; Universite du Quebec a Trois-Rivieres, CANADA

## Abstract

Penicillin binding proteins (PBPs) are normal constituents of bacterial which are absent in mammalian cells. The theoretical binding modes of known oxazin-5-ones toward the protein were used as a guide to synthesis new inhibitors. Structural studies of protein-ligand complexes revealed that conformational discrepancies of the derivatives in the protein’s binding site gave rise to the variation in their inhibition constant which ranged from 68.58 μM to 2.04 mM. Biological assay results further confirmed the antibiotic potencies of the studied compounds. Although the outcome of biological screening does not parallel computational predictions, the results obtained from both methods suggest that the oxazin-5-one derivatives are potential PBP inhibitors, hence interesting antibiotic lead agents.

## Introduction

Phenoxazine and its derivatives like phenothiazine compounds are important class of nitrogen-oxygen heterocyclic compounds that were widely used as dyes and pigments [1–3] but have been found, recently, to exhibit broad spectrum of pharmacological activity such as CNS depressant, sedatives, antiepileptics, herbicidal, tranquilizers, antituberculosis, antitumor, antibacterial, spasmolytic, anthelminthes and parasitical effects [4–8]. Wesolowska *et al* have recently reported the biological activities of newly synthesized water soluble 2-amino-4,4α,7-dimethyl-3*H*-phenoxazin-3-one [9]. In view of the foregoing, our research group has been focusing on finding novel antimicrobial leads in phenothiazine and phenoxazine derivatives [10–14].

Nowadays, computational methods are routinely employed in drug development processes due to their reliability, time and cost effectiveness [15–17]. These methods involve; calculation of pharmacokinetic parameters of chemical compounds using molecular descriptors, pharmacophore screening, docking and binding free energy calculations of a given interaction. Information derived from the binding mode of docked compound has been employed as a guide in structural optimization processes. [18–19].

In the present work, we used results of binding mode predicted from docking calculations of two parent molecules to guide the synthesis of new oxazin-5-ones via palladium catalyzed cross-coupling 6-chloro-5*H*-benzo[a]phenoxazin-5-one and 6-chloro-*5H*-naphtho[2,1-b]pyrido[2,3-e][1,4]oxazin-5-one with boronic acids, terminal alkynes and organotin reagents. The lowest theoretical free energy of binding of the derivatives toward a validated anti-bacterial drug target (penicillin binding protein) was determined by docking calculations and their predicted binding mode gave basis for the various observed interactions. Finally, biological assay of the derivatives against selected clinical bacterial confirmed their antibiotic potency. Although the results of the two screening methods do not exactly tally, our findings suggest that some of the analogues could indeed serve as leads in designing novel antibiotic drugs.

## Results and Discussion

Penicillin binding proteins (PBPs) are normal constituents of bacterial. They are known as PBPs because of their high affinity for penicillin. One of the PBPs considered in this study is DD-transpeptidase (DDTP). DDTP is involved in the biosynthesis of bacterial cell wall by catalyzing the transfer of R-Laca-D-alanyl moiety of R-L-aca-D-alanyl-D-alanine carbonyl donors to gamma OH of their active site serine and from this to a final acceptor [20–21]. Therefore, DDTP is essential for the maintenance of bacterial cell wall integrity and ultimately bacterial survival. This enzyme is a drug target of choice in chemotherapy of bacterial infection for two reasons: it is accessible from the periplasm and has no equivalent in mammalian cells [22].

**Fig 1:**
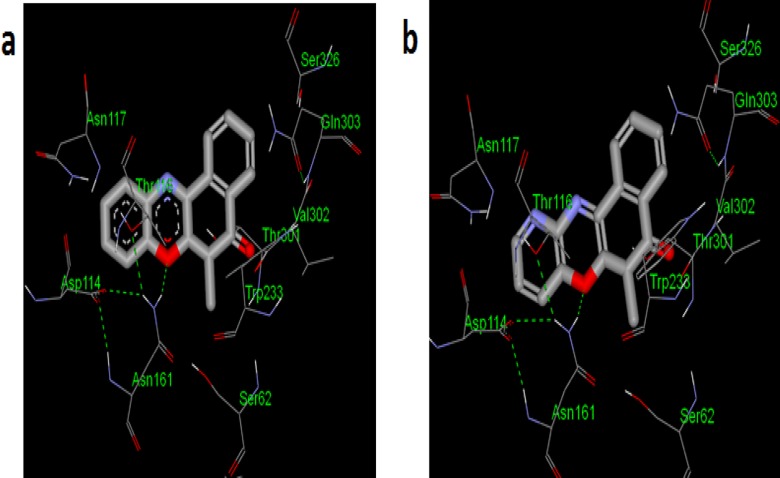
Docked poses of (a) 1 and (b) 2 toward the binding site cavity of PBP.

It was observed that compounds **1** and **2** demonstrated the following; affinity for the studied PBP at inhibition constants (*K*_i_) of 169.76 and 383.47 μM respectively, low molecular weights—MW (281.70 and 282.69 respectively), low total polar surface area—TPSA (43.10 and 55.99 respectively), and reasonable ligand efficiencies (0.26 and 0.23 respectively). Therefore, compounds **1** and **2** were considered as good starting hits for structural modification. Furthermore, compounds **1** and **2** were assayed against *Bacillus cereus*, *Staphylococcus aureus*, *Escherichia coli* and *Pseudomonas aeruginosa* and were found to have inhibition zone diameters (IZDs) of 6 and 21, 7 and 15, 10 and 6, 6 and 4 respectively. Analysis of compounds **1** and **2** binding mode within the active site of PBP (Fig 1A and 1B) suggests that substitution of the chlorine atom at 6-position with lipophilic and/or an extended hydrophilic moieties could lead to improved potency due to the presence of TRP233 and SER62 residues.

### Synthesis of the Oxazin-5-ones

**Fig 2:**
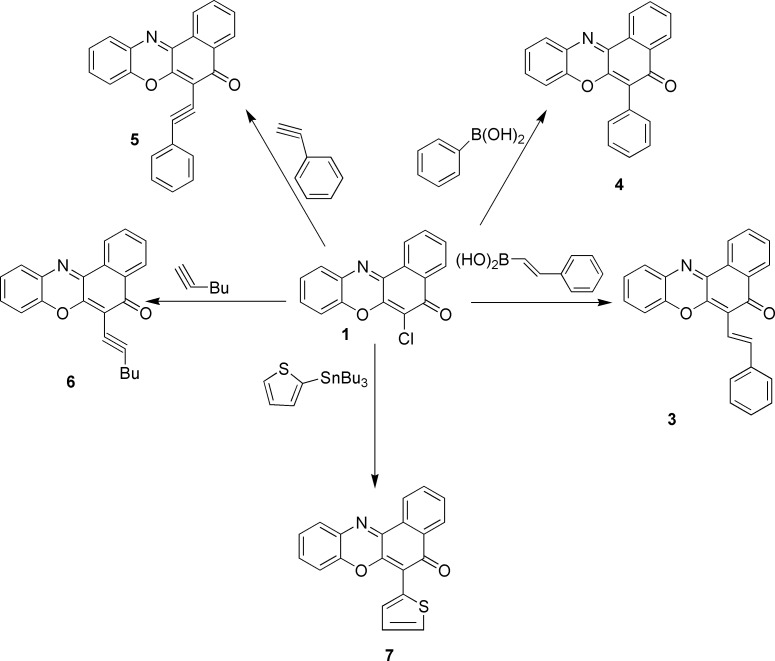
Reaction conditions and reagents: Ar-Cl (1 equiv.), boronic acid (1.2 equiv.)/ alkyne (1.5 equiv.), K_3_PO_4_ (3mM), CH_3_CN–H_2_O (2:1) (3 mL). For Sonogashira and Stille reactions CH_3_CN (3 mL) was used instead of CH_3_CN–H_2_O (2:1) (3 mL). Compounds were purified by flash column chromatography.

The intermediates, 6-chlor-5*H*-benzo[a]phenoxazin-5-one **1** and 6-chloro-*5H*-naphtho[2,1-b]pyrido[2,3-e][1,4]oxazin-5-one **2** were prepared by anhydrous base catalysed coupling of 2,3-dichloro-1,4-naphthoquinone with 2-aminophenol and 2-aminopyridinol respectively at room temperature[12–14]. The styrylation and arylation of 6-chloro-5*H*-benzo[a]phenoxazin-5-one **1** via Suzuki-Miyaura protocol supplied (*E*)-6-Styryl-5*H*-benzo[a]phenoxazin-5-one, **3** and 6-phenyl-5*H*-benzo[a]phenoxazin-5-one **4** respectively in good yields. Compounds 6-(phenylethynyl)-5*H*-benzo[a]phenoxazin-5-one**5** and 6-(hex-1-yn-1-yl)-5*H*-phenoxazin-5-one**6** were correspondingly obtained by Pd (0)/Xphos mediated alkynylation of compound **1**. In another reaction 6-chlor-5*H*-benzo[a]phenoxazin-5-one **1** was coupled with tributylthiophenylstannane to afford 6-(thiophen-2-yl)-5*H*-benzo[a]phenoxazin-5-one7 (Fig 2).

**Fig 3:**
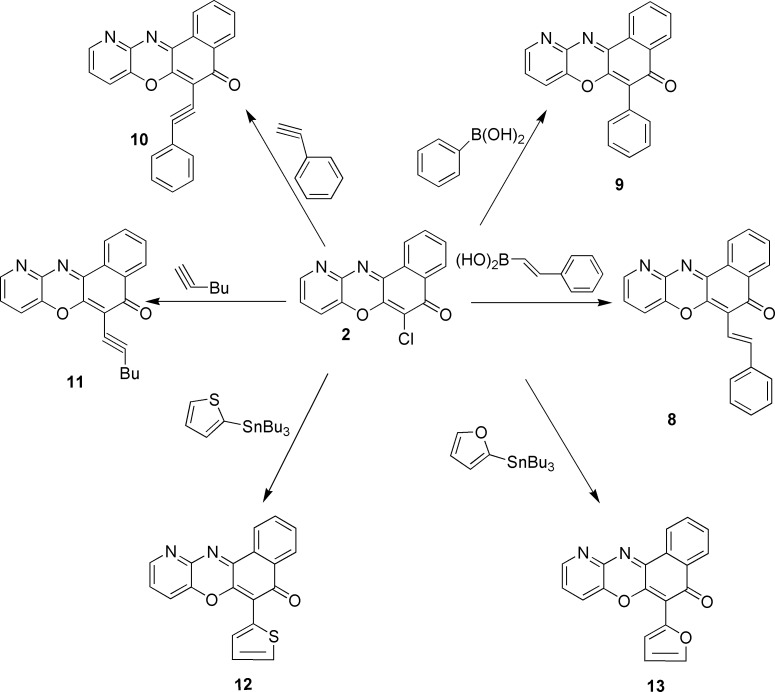
Reaction conditions are the same as in Fig 2.

Similarly, the cross-coupling of 6-chloro-5*H*-naphtho[2,1-b]pyrido[2,3-e][1,4]oxazin-5-one **2** with styryl and aryl boronic acids gave compounds (*E*)-6-styryl-5*H*-naphtho[2,1-b]pyrido[2,3-e][1,4]oxazin-5-one, **8** and 6-phenyl-5*H*-naphtho[2,1-b]pyrido[2,3-e][1,4]oxazin-5-one, **9** as reddish and pinkish coloured solid respectively. 6-(Phenylethynyl)-5H-naphtho[2,1-b]pyrido[3,2-e][1,4]oxazin-5-one,**10** and 6-(Hex-1-yn-1-yl)-5H-naphtho[2,1-b]pyrido[3,2-e][1,4]oxazin-5-one, **11** were obtained in high yields by reaction of ethynyl benzene and hex-1-yne with 6-chloro-5*H*-naphtho[2,1-b]pyrido[2,3-e][1,4]oxazin-5-one **2** respectively. In another reaction compound **2** was coupled with thiophenylstannane and furanylstannane to supplied compounds 6-(thiophen-2-yl)-5*H*-naphtho[2,1-b]pyrido[3,2-e][1,4]oxazin-5-one **12** and 6-(furan-2-yl)-5*H*-naphtho[2,1-b]pyrido[3,2-e][1,4]oxazin-5-one **13** respectively (Fig 3). The structures of the synthesized compounds were established by spectral and elemental analytical data.

### Docking Calculations

To probe the C6 position, compounds **3** to **13** were synthesized (Figs 2 and 3) and docked toward PBP binding site. It was observed that compounds **4**, **9**, **7** and **12** inhibited the activity of the studied target (*K*_i_ values ranging from 96.31 to 68.58 μM) (Table 1) more than compounds **1** and **2**, but their relatively poor ligand efficiencies (21–23 kcal/mol per non-H atom) could pose a challenge [23]. Activities were greatly reduced in other derivatives with compound **10** having *K*_i_ as poor as 2.04 mM.

**Table 1:** Docking calculations of the compounds toward PBP.

Comp. code	PBP		
	ΔG (Kcal/mol)	*K*_i_ (μM)	ligand efficiency
**3**	-4.45	546.24	0.16
**4**	-5.66	71.54	0.23
**5**	-4.13	931.82	0.15
**6**	-4.34	661.71	0.19
**7**	-5.48	96.31	0.23
**8**	-4.16	893.7	0.15
**9**	-5.71	68.58	0.21
**10**	-3.67	2.04*	0.23
**11**	-4.50	503.41	0.19
**12**	-5.49	94.55	0.23
**13**	-4.94	239.54	0.21

The * values are in mM unit

### Assessment of Oral Bioavailability Property

Criteria proposed by Lipinski in his popular “rule of five” (ro5) alongside total polar surface area (TPSA) property were used to assess the oral bioavailability potential of the newly synthesized oxazin-5-ones [24]. Total polar surface area (TPSA) is frequently used in drug design as surrogate property for cell permeability with a rule-of-thumb that a molecule with a TPSA of less than 140 Å^2^ would be able to permeate the cell. TPSA has also been used as a surrogate for penetrating the blood-brain-barrier (BBB). Van de *et al* [25] demonstrated that for a drug molecule to cross the central nervous system, the cut-off for TPSA should be ≤ 90 Å^2^. This implies that all compounds can penetrate blood-brain barriers, hence can be used in treating brain cells infections.

According to Lipinski’s ro5, derived from 90^th^ percentile of drug candidates that reached phase II clinical trials, to be drug-like, a drug candidate should have lipophilicity (log P) ≤ 5, molecular weight (MW) ≤ 500, number of hydrogen bond acceptor (HBA) ≤ 10, and number of hydrogen bond donor (HBD) ≤ 5. The rule claims that drug candidate which violates more than one property will have bioavailability problem. Table 2 showed that all the compounds are drug-like with respect to ro5. Veber *et al* [26] observed number of rotatable bond (NRB) experimentally influences bioavailability in rats. Therefore, NRB ≤ 10 has been recommended for good oral bioavailability property. Again all the compounds respected NRB criteria for drug-likeness.

**Table 2:** Physicochemical properties for drug-likeness.

Comp	MW	Log P	HBD	HBA	nViolation	TPSA	NRB
**1**	281.70	4.36	0	3	0	43.10	0
**2**	282.69	3.46	0	4	0	55.99	0
**3**	349.39	6.06	0	3	1	43.10	2
**4**	323.35	5.47	0	3	1	43.10	1
**5**	347.37	5.11	0	3	1	43.10	0
**6**	327.38	5.82	0	3	1	43.10	2
**7**	329.38	5.26	0	3	1	43.10	1
**8**	350.38	5.16	0	4	1	55.99	2
**9**	324.34	4.58	0	4	0	55.99	1
**10**	348.36	4.21	0	4	0	55.99	0
**11**	328.37	4.92	0	4	0	55.99	2
**12**	330.37	4.36	0	4	0	55.99	1
**13**	314.30	3.72	0	5	0	55.99	1

MW = molecular weight; Log P = partition coefficient; HBD = no. of hydrogen bond donor; HBA = no. of hydrogen bond acceptor; TPSA = total polar surface area; NRB = no. of rotatable bond; nViolation = no. of Lipinski violation.

### Binding Mode Prediction

The docked poses of all the derivatives toward PBP binding site is shown in Part 4a in S1 Fig. It was observed that the compounds adopted varying preferential conformations within the PBP binding groove, which might have orchestrated the discrepancies observed in their affinities for the protein. The four derivatives which inhibited PBP activity more than compounds **1** and **2** have similar binding modes and the interactions of the moieties attached at their C6 position with the protein residues apparently account for their greater affinity for PBP than the rest of the derivatives. Part 4b in S1 Fig showed that a strong hydrophobic interaction exist between compounds **4** and **9** phenyl groups at C6 position and TYR159 phenyl group and PHE120 alkyl side chain of the protein. The poses of compounds **7** and **12** suggest that they made strong polar contacts with ASN161 using their thiophenyl moieties at C6 position. Also, the additional hydrogen bonding between compounds **9** and **12** hetero nitrogen and TYR306 might account for the improved potencies (68.58 and 94.55 μM respectively) over compounds **4** and **7** (71.54 and 96.31 μM respectively). Docking calculations showed that compounds **3**, **5**, **8** and **10** have similar binding poses which are different from that of compounds **1** and **2** (Part 4c in S1 Fig). Their styryl and phenylethynyl moieties were accommodated within the protein groove surrounded by PHE120, VAL302, GLN303, and LEU214 residues. Double bonds are longer than triple bonds. Therefore, compounds **3** and **8** docked deeper into the PBP hydrophobic pocket and hence, made a stronger interactions (546.24 and 893.70 μM respectively) than compounds **5** and **10** (931.82 μM and 2.04 mM respectively). In fact, it appeared the ability to dock deep into PBP binding cavity is a necessary criteria for interaction with the protein because the better inhibitory activities of compounds **6** and **11** than those of compounds **5** and **10** could be attributed to the length of compounds **6** and **11** hexynyl substituent (Part 4d in S1 Fig). In general, the derivatives demonstrated no significant improved affinity for the studied target over the known oxazines (compounds **1** and **2**).

### Biological Screening

All the derivatives were screened *in vitro* against selected bacterial following Bauer *etal* method [27] and results are shown in Table 3. In general, the synthesized derivatives manifested appreciable activity but not in consonant with the docking calculation results. Perhaps PBP was not the drug target inhibited by the derivatives in the whole cell assays and hence the variation in their results.

**Table 3:** Antimicrobial Evaluation of 6-chloro-5*H*-benzobenzo[a]phenoxazin-5-one and 6-chloro-5*H*-naphtho[2,1-b]pyrido[3,2-e][1,4]oxazin-5-one and their derivatives determine by diffusion method. Inhibition zone diameter (mm). Minimum inhibition concentration (μg/mL) in bracket ().

compound	Antibacterial activity			
	Gram positive		Gram negative	
	B.c	S.a	E.c	P.a
1	6	7	10 (14.1)	6
2	21(5.6)	15(10.2)	6	4
3	-	-	-	-
4	-	-	-	-
5	20(5.00)	22(5.12)	4	3
6	12(10.0)	12(16.6)	-	-
7	10(17.0)	9	6	24(7.4)
8	12 (21.0)	17(11.5)	8	-
9	7	16(10.2)	-	5
10	17(3.2)	18(5.50)	7	-
11	5	16(10.0)	5	-
12	20(10.0)	12(21.0)	14(16.2)	20(5.0)
13	18(9.0)	23(7.9)	7	22(10.5)
Tetracycline	29 (4.0)	41 (5.0)	24 (5.0)	20 (1.0)

B. c (*Bacillus cereus*); S. a (*Staphylococcus aureus*); E. c (*Escherichia coli*); P. s (*Pseudomonas aeruginosa*); Conc. = μg/mL of DMSO

Apart from compounds **3** and **4,** the synthesized derivatives exhibited activity against both Gram-positive and Gram-negative bacteria. With exception of compounds **7**, **12** and **13** the rest compounds appeared to be generally more active for Gram-positive than Gram-negative bacteria. The compounds (**8**–**13)** derived from 6-chloro-5*H*-naphtho[2,1-b]pyrido[3,2-e][1,4]oxazin-5-one whose structure contain nitrogen hetro-atom in position-10 of the molecule exhibited enhanced activity compared to those derived from 6-chloro-5*H*-benzo[a]phenoxazine substrate which has no *N*-hetero-atom at position 10. Compounds **12** and **13** particularly seem to have broad activity for Gram-positive and Gram-negative bacteria and this was attributed partly to heterocyclic thiophenyl and furanyl moieties contained in the molecules. In addition, the MICs of the compounds were higher than the reference drugs. However, the MIC of compounds **5** and **10** are close to that of tetracycline for *B*. *cereus* and *S*. *aureus* bacteria respectively.

It can be observed in this study that the results of biological assay and *in silico* screening do not parallel. This is often the case when comparing the results of *in-silico* screening, which focuses on a particular enzyme, with a whole organism *in vitro* testing. The reason could be that the enzyme used in the *in silico* study might not be *in vitro* mechanism of the drug candidate action [28].

## Experimental Section

### General Information

All chemicals were purchased from Aldrich Chemical Company UK and were used without further purification. Otherwise stated all compounds were synthesized and characterized in the School of Chemistry of Cardiff University UK. Melting points was determined with a Fischer-Johns apparatus. ^1^H and ^13^C NMR data were recorded with Brucker DPX 400 MHz spectrometers relative to TMS as internal standard. All and chemical shifts reported in ppm (δ) and coupling constants (*J*), reported in Hz. Multiplicity is indicated using the following abbreviations: br, for broad; s, for singlet; d, for doublet; t, for triplet; dd, for doublet of doublets and; m, for multiplet. The mass spectra data were obtained on a Varian 1200 Quadruple Mass and Micromass Quadro II Spectrometers. Elemental Analysis was carried out with Thermo Quest Flash 1112 series (CHNS) Elemental Analyser. UV-Visible spectra were recorded on Cecil 7500 Aquarius 7000 Series Spectrometer at Chemistry Advance Laboratory (CAL), Sheda Science & Technology Complex (Shestco) Abuja, Nigeria, using matched 1cm quartz cells and methanol as solvent. The absorption maxima are recorded in nanometers (nm) and figures in parenthesis are log ε. Microorganisms were obtained from Bishop Shahanan Hospital, Nsukka, Enugu State while the antimicrobial evaluations were carried out in Faculty of Pharmaceutical Sciences, University of Nigeria, Nsukka, Nigeria.

### Molecular Modeling

The x-ray crystal structure of PBP (DDTP) with its co-crystallized inhibitor was retrieved from protein data bank (PDB code 1CEF) [20]. Molecular operating environment (MOE) was used to treat the complex dimers as described in our earlier work [29] and to generate the three dimensional structures of the benzophenoxazines.

AutoDock 4.2.0 was employed to perform the docking calculations [30]. A grid box size of 22 24 24 A^3^ points (spacing between the grid points of 0.375 A) was used which centered on the mass center (20.472, -11.823, 40.666) of the crystallographic macromolecule encompassing all active site atoms. The docking protocol was validated by calculating the root mean square deviation of the docked ligand from the x-ray crystallized ligand.

The MW, NRB, log P, HBA, HBD, number of Lipinski violations and TPSA were calculated using MolinspirationChemoinformatics software 2016.

#### General Procedure I (Suzuki Cross-Coupling Reactions)

To an oven dried 10 mL RB flask containing 2 mL of CH_3_CN and 1 mL of water was added RX (1 mmol), RB(OH)_2_ (1.2 mmol), K_3_PO_4_ (588mg, 3 mmol) and the reaction mixture gradually warm to 40°C while stirring under nitrogen atmosphere. Pd(OAc)_2_ (8.92mg, 4 mol%), X**-**Phos (32.5mg, 7 mol%) were added and reaction vessel cork with rubber septum. The entire reaction mixture was heated at 80°C within 5–8 h, and then cooled to room temperature. Solvent evaporated in vacuum and crude product extracted from water with DCM (10 mL x 4). The combined organic extracts were dried with MgSO_4_ and concentrated in vacuum. Crude product was purified by flash column chromatography on silica gel.

#### General Procedure II (Sonogashira Cross-Coupling reactions)

Acetonitrile (3 mL) was degassed for 0.5 h before injection into an oven-dried 10 mL RB flask fitted with a rubber septum already charged with Pd(OAc)_2_ (8.9 mg, 4 mol%), X**-**Phos (32.5mg, 7 mol%), RX (1mmol) and K_3_PO_4_ (588mg, 3mmol), under an atmosphere of nitrogen. The reaction mixture was stirred and warmed to 50°C during which time 1-alkyne (1.5 mmol) was gradually injected *via* syringe. The reaction temperature was maintained for 0.5 h before being increased to 80°C. Stirring was continued for 5–8 h then the mixture was cooled to room temperature after reaction completion as monitored by TLC. Water (10 mL) was added mand product extracted with dichloromethane (4 x 10 mL). The combined organic extracts were dried (MgSO_4_) and concentrated in vacuum. The crude product was separated by flash chromatography on silica gel using petroleum ether**-** ethyl acetate mixtures.

#### General Procedure III (Stille Cross-Coupling Reactions)

An oven-dried 10 mL RB flask was charged with Pd(OAc)_2_ (8.92 mg, 4 mol%) and X-Phos (32.5 mg, 7 mol%) and covered with rubber septum. The vessel was evacuated and back-filled with N_2_ thrice before injecting of CH_3_CN (2mL) and H_2_O (1 mL) (both solvents degassed for 30 min) and the reaction mixture warmed to 50°C within 10 min. Rubber septum quickly removed to add chlorophenothiazine (1mmol) and K_3_PO_4_ (318 mg, 1.5 mmol), and replaced before injecting tributylthienylstannane or tributylfuranylstannane (1.2 mmol). The temperature was gradually increase to and maintained 80°C. The reaction was terminated in 5 h and the crude product extracted from water (10 mL) four times with DCM. The combined organic extract was dried with MgSO_4_ and concentrated in vacuum. The crude product was purified by flash chromatography on silica gel using petroleum ether- ethyl acetate eluent.

#### (*E*)-6-Styryl-5*H*-benzo[a]phenoxazin-5-one (3) (Fig 2)

General procedure **I** was applied in the conversion of styrylboronic acid and 6-chlorobenzo-5*H*-phenoxazin-5-one to the title product 8 h. Analytical pure product was obtained by flash column chromatography using 10% EtOAc/ 90% pet. ether eluent as dark purple solid. Yields = 179 mg (51%). M pt. 185–187°C. δ_H_ (400 MHz, CDCl_3_): 8.60–8.58 (1H, m); 8.24–8.22 (1H, m); 8.04**–**8.00 (1H, d, J = 8.02); 7.74–7.63 (3H, m); 7.56–7.48 (3H, m); 7.44–7.38 (3H, m); 7.34**–**7.22(3H, m). δ_c_ (150 MHz, CDCl_3_,: 182.88 (carbonyl carbon, C = O), 147.13, 146.62, 144.21, 138.45, 136.34, 133.12, 131.99, 131.72, 131.19, 130.97, 129.66, 128.10, 126.96, 126.37, 125.42, 124.47, 117.73, 115.79, 114.98. UV**-**Visible λ_max_ (MeOH): 287.5 (4.81); 313.0 (4.01); 491.0 (3.43); 643.5(4.01); 737 (3.98); 809 (3.10). MS (AP), m/z (% relative intensity): 90.01(9), 92.01(5), 116.06(5), 350.11[(100), M^+^**-**1], 351.11[(30), M^+^**-** 2]. Anal.calcd. for C_24_H_15_NO_2_: C, 82.51; H, 4.33; N, 4.01. Found: C, 82.21; H, 4.53; N, 4.21.

#### 6-Phenyl-5*H*-benzo[a]phenoxazin-5-one (4) (Fig 2)

The general procedure **I** was used to convert phenyl boronic acid and 6-chloro-5*H*-benzo[a]phenoxazin-5-one to provide the title product 7 h. Purification by flash column chromatography (10% EtOAc/ 90% pet. ether eluent) supplied the analytical pure product as orange solid. Yields = 174 mg (54%). M pt. > 219°C (dec.). δ_H_ (400 MHz, CDCl_3_): 8.69–8.67 (1H, d, J = 8.68); 8.28–8.27 (1H, d, J = 8.27); 7.74–7.67 (3H, dd, J = 7.71, 1.12); 7.45–7.43 (4H, d, J = 7.44); 7.35–7.33 (2H, d, J = 7.37); 7.08–7.06 (1H, d, J = 7.07). δ_c_ (150 MHz, CDCl_3_: 182.37 (carbonyl carbon, C = O), 147.39, 146.83, 144.11, 132.84, 132.03, 131.98, 131.83, 131.24, 131.04, 130.91, 130.68, 129.58, 128.07, 127.97, 126.49, 125.14, 124.51, 119.38, 116.01.UV**-**Visible λ_max_ (MeOH): 309.0 (8.87); 445.0 (8.96); 749 (6.60). MS(AP), m/z (% relative intensity): 324.12[(100), M^+^**-** 1], 325.13 [(25), M^+^**-** 2]. Anal. cacld. for C_22_H_13_NO_2_: C, 81.72; H, 4.05; N, 4.33. Found: C, 81.91; H, 4.16; N, 4.21.

#### 6-(Phenylethynyl)-5*H*-benzo[a]phenoxazin-5-one (5) (Fig 2)

The general procedure II was used to couple ethynylbenzene and 6-chloro-5*H*-benzo[a]phenoxazin-5-one within 7 h. Purification by flash chromatography (10% EtOAc/pet. ether) gave the pure product **5** as a dark red solid. Yield = 208 mg (60%). M.p. >110°C (dec). δ_H_ (400 MHz, CDCl_3_): 8.62 (1H, d, *J* = 8.6); 8.26 (1H, d, *J* = 7.2); 7.77–7.60 (5H, m); 7.44–7.31 (6H, m). δ_c_ (150 MHz, CDCl_3_): 181.6 (C = O), 151.9, 147.0, 144.6, 133.7, 132.8, 132.4, 132.4, 132.1, 131.2, 130.3, 129.2, 128.8, 126.9, 126.2, 125.2, 123.6, 116.7, 103.1 (alkynyl carbon), 81.0 (alkynyl carbon). UV**-**Visible λ_max_ (MeOH): 337.5 (3.26); 352.5(4.15); 468.5 (4.14); 748.0 (6.79). MS(APCI), m/z(% relative intensity): 275 (5), 348 [(100), M^+^**+**1]. Anal.calcd. for C_24_H_13_NO_2_: C, 82.98; H, 3.77; N, 4.03. Found: C, 83.01; H, 3.79; N, 4.16.

#### 6-(Hex-1-yn-1-yl)-5*H*-benzo[a]phenoxazin-5-one (6) (Fig 2)

The general procedure II was used to couple hex-1-yne and 6-chloro-5*H*-benzo[a]phenoxazin-5-one **2** to give the title product in 8 h. Purification by flash chromatography (10% EtOAc/pet. ether) provided analytically pure compound **6** as an orange solid. Yield = 121 mg (37%). M.p. > 70°C (dec). δ_H_ (400 MHz, CDCl_3_): 8.64–8.62 (1H, m, Ar-H); 8.27–8.25 (1H, m, Ar-H); 7.78–7.66 (3H, m, Ar-H); 7.46–7.42 (1H, m, Ar-H); 7.33–7.29 (2H, m, Ar-H); 2.57 (2H, t, -CH_2_- *J* = 7.1); 1.67–1.52 (4H, m, -CH_2_-CH_2_-); 0.95 (3H, t, CH_3_-, *J* = 7.1). δ_c_ (150 MHz, CDCl_3_): 181.9 (C = O), 147.0, 144.5, 133.4, 132.4, 132.1, 131.8, 131.7, 131.0, 130.0, 126.6, 125.8, 124.9, 116.4(alkynyl carbon), 105.16(alkynyl carbon), 30.9, 29.9, 22.2, 20.3 and 13.9. UV-Visible λ_max_ (MeOH): 360.0 (4.11); 464.0 (3.84); 750.0 (3.97). MS (APCI), m/z(% relative intensity): 286 (10), 301 (10), 328 [(100), M^+^**-**1]. Anal.calcd. for C_22_H_17_NO_2_: C, 80.71; H, 5.23; N, 4.28. Found: C, 81.74; H, 5.21; N, 4.31.

#### 6-(Thiophen-2-yl)-5*H*-benzo[*a*]phenoxazin-5-one (7) (Fig 2)

Procedure III was used to prepare the title product from the cross-couping of tributyl 2-thienyl stannane with 6-chloro-5*H*-benzo[*a*]phenoxazin-5-one in 6 h. Purification by flash chromatography applying 10% EtOAc/ 90% pet. ether as eluent provided the analytically pure dark brown solid product. Yield = 240mg (73%). Mp = 203–204°C. NMR: δ_H_ (400 MHz, CDCl_3_): 8.57–8.65 (1H, m); 8.33**–**8.31 (1H, m); 8.22–8.20 (1H, dd, *J* = 8.21, 8.21); 7.79–7.77 (1H, dd, *J* = 7.74, 1.74); 7.72–7.68 (2H, m); 7.52–7.50 (1H, dd, *J* = 7.51–1.47); 7.47–7.39 (2H, m); 7.34–7.30 (1H, m); 7.19–7.16 (1H, m). δ_c_ (150 MHz, CDCl_3_): 181.91 (C = O), 146.85, 145.05, 143.85, 132.89, 132.09, 131.81, 131.78, 131.67, 131.35, 130.07, 129.60, 128.63, 126.62, 126.22, 125.55, 124.43, 115.85, 112.73. UV-Visible λ_max_ (MeOH): 364.5 (4.08); 493.0 (3.64); 750.0 (4.02). HRMS (EI), *m/z* (% relative intensity): 83.9533 (100), 142.5381 (8), 272.0522 (10), 301.0556 (12), 329.0512 [(93), M^+^]. Anal.calcd. for C_20_H_11_NO_2_S: C, 72.93; H, 3.37; N, 4.25; S, 9.73. Found: C, 72.98; H, 3.40; N, 4.31; S, 9.54.

#### (*E*)-6-Styryl-5*H*-naphtho[2,1-b]pyrido[2,3-e][1,4]oxazin-5-one (8) (Fig 3)

General procedure I was applied in the conversion of styrylboronic acid and 6-chlorobenzo-5*H*-naphtho[2,1-b]pyrido[2,3-e]oxazin-5-one to the title product in 8 h. Purification by flash column chromatography (40% EtOAc/ 60% pet. ether eluent) gave the analytical pure product as dark purple solid. Yields = 228 mg (65%). M pt.: > 170°C (dec). δ_H_ (400 MHz, CDCl_3_): 8.75–8.73 (1H, dd, J = 8.74, 1.64); 8.53–8.51 (1H, dd, J = 8.52, 1.73); 8.25–8.22 (1H, dd, J = 8.24, 1.17); 8.05–8.01 (1H, d, J = 8.03); 7.71–7.65 (3H, m); 7.55–7.53(2H, d, J = 7.54); 7.44–7.40 (1H, d, J = 7.42); 7.37–7.30 (3H, m); 7.26–7.24(1H, m). δ_c_ (150 MHz, CDCl_3_): 182.85 (carbonyl carbon, C = O), 151.41, 146.71, 145.58, 145.03, 138.07, 137.73, 132.72, 132.49, 131.93, 128.75, 128.50, 127.07, 126.52, 125.56, 125.47, 124.05, 117.25. UV**-**Visible λ_max_ (MeOH): 306.0 (8.99); 450.0 (7.65); 749.5 (6.87). MS (AP), m/z(% relative intensity): 90.01(36), 92.01(12),116.07(6), 351.11[(100), M^+^**-** 1], 352.11[(25), M^+^**-** 2]. Anal.calcd. for C_23_H_14_N_2_O_2_: C, 78.84; H, 4.03; N, 8.00. Found: C, 79.02; H, 4.15; N, 8.14.

#### 6-Phenyl-5*H*-naptho[2,1-b]pyrido[2,3-e][1,4]oxazin-5-one (9) (Fig 3)

The general procedure was used to convert phenyl boronic acid and 6-chloro-5*H*-naphtho[2,1-b]pyrido[2,3-e][1,4]oxazin-5-one to the title product in 8 h. Analytical pure product was obtain by flash column chromatography (40% EtOAc/60% pet. ether eluent) as red solid. Yield = 143 mg (44%). M pt.: > 238°C (dec). δ_H_ (400 MHz, CDCl_3_): 8.88–8.85 (1H, m); 8.53–8.51 (1H, dd, J = 8.52, 1.72); 8.31–8.29 (1H, m); 7.78–7.75 (2H, m); 7.47**–**7.44 (5H, m); 7.40–7.37(1H, m), 7.32–7.29(1H, dd, J = 7.31). δ_c_ (150 MHz, CDCl_3_): 182.41 (carbonyl carbon, C = O), 151.75, 146.43, 145.95, 144.64, 140.91, 135.60, 132.94, 132.56, 131.91, 130.66, 130.55, 130.30, 129.56, 128.48, 128.33, 128.09, 127.56, 127.27, 126.65, 125.77, 125.57, 124.44, 120.63, 115.45. UV-Visible λ_max_ (MeOH): 266.0(8.96); 275.5 (8.83), 360.0 (8.63); 446.0 (8.15); 753.0 (6.55). MS (EI), m/z(% relative intensity): 83.9524(65), 162.0445(5), 214.0643(5), 240.0855(5), 266.0854(8), 295.0894(20), 312.1321(10), 323[(100), M^+^ + 1]. Anal. calcd. for C_21_H_12_N_2_O_2_: C, 77.77; H, 3.73; N, 8.64. Found: C, 77.52; H, 3.85; N, 8.74.

#### 6-(Phenylethynyl)-5*H*-naphtho[2,1-b]pyrido[3,2-e][1,4]oxazin-5-one (10) (Fig 3)

The general procedure was used to coupled ethynylbenzene with 6-chloro-5*H*-naphtho[2,1-b]pyrido[3,2-e][1,4]oxazin-5-one**5** within 8 h. The pure compound **10** was obtained as a dark reddish solid after flash chromatography (40% EtOAc/pet. ether). Yield = 233 mg (67%). M.p. > 190°C (dec). δ_H_ (400 MHz, CDCl_3_): 8.81**–**8.78 (1H, m), 8.58 (1H, dd, *J* = 8.5, 2.1); 8.28–8.26 (1H, m); 7.76–7.72 (3H, m); 7.62–7.58 (2H, m); 7.41 (1H, dd, *J* = 7.4, 1.9); 7.34–7.31 (3H, m). δ_c_(150 MHz, CDCl_3_): 181.0 (C = O), 150.9, 150.6, 147.4, 145.1, 141.2, 133.1, 133.0, 132.2, 131.6, 130.5, 129.3, 128.6, 126.8, 126.2, 125.9, 124.8, 123.0, 105.8 (alkynyl carbon), 104.1 (alkynyl carbon). UV-Visible λ_max_ (MeOH): 360.5(3.89); 479.0 (3.74); 748.0 (4.67). MS(APCI), m/z(% relative intensity): 349 [(100), M^+^**+**1], 350 [(37), M^+^**+**2]. Anal. calcd. for C_23_H_12_N_2_O_2_: C, 79.30; H, 3.47; N, 8.04. Found: C, 79.47; H, 3.65; N, 8.16.

#### 6-(Hex-1-yn-1-yl)-5*H*-naphtho[2,1-b]pyrido[3,2-e][1,4]oxazin-5-one (11) (Fig 3)

The general procedure was used to convert hex-1-yne and 6-chloro-5*H*-naphtho[2,1-b]pyrido[3,2-e][1,4]oxazin-5-one**5**into the title product in 8 h. Purification by flash chromatography employing 40% EtOAc/pet. ether gave analytically pure oxazin-5-one **11** as a dark red solid. Yield = 69 mg (21%). M. p. > 81°C (dec). δ_H_ (400 MHz, CDCl_3_): 8.80–8.78 (1H, m); 8.57 (1H, dd, *J* = 8.6, 1.9); 8.27–8.25 (1H, m); 7.76–7.67 (3H, m); 7.40 (1H, dd, *J* = 7.4, 1.9); 2.58 (2H, t, -CH_2_-, *J* = 7.0); 1.65–1.52 (4H, m, -CH_2_-CH_2_-); 0.94 (3H, t, -CH_3_, *J* = 7.1). δ_c_ (150 MHz, CDCl_3_): 180.3 (C = O), 150.0, 149.7, 145.9, 144.0, 140.2, 131.8, 131.7, 130.6, 129.4, 125.6, 124.8, 124.7, 123.4, 105.5 (alkynyl carbon), 105.4 (alkynyl carbon), 70.3, 29.6, 28.7, 21.0, 19.1, 12.6. UV-Visible λ_max_ (MeOH): 355.0 (3.41); 464.0 (3.34); 751.0 (3.87). MS (EI), m/z (% relative intensity): 83.9476 (100), 207.0321 (12), 221.0739(12), 282.0200(15). Anal.calcd. for C_21_H_16_N_2_O_2_: C, 76.81; H, 4.91; N, 8.53. Found: C, 76.95; H, 5.08; N, 8.61.

#### 6-(Thiophen-2-yl)-5*H*-naphtho[2,1-*b*]pyrido[2,3-*e*][1,4]oxazin-5-one (12) (Fig 3)

Procedure III was used to cross-couple tributyl 2-thienyl stannane with 6-chloro-5*H*-naphtho[2,1-*b*]pyrido[2,3-*e*][1,4]oxazin-5-oneto afford the title product within 6 h. Purification by flash chromatography (45% EtOAc/ 55% pet. ether eluent) gave the analytically pure dark brown solid product. Yield = 257 mg (78%). M p. > 210°C (dec). NMR: δ_H_ (400 MHz, CDCl_3_): 8.76**–**8.74 (1H, m); 8.55–8.53 (1H, dd, *J* = 8.54, 8.54); 8.26–8.24 (1H, m); 8.14–8.13 (1H, dd, *J* = 8.14); 7.72–7.67 (2H, m); 7.51–7.49 (1H, dd, *J* = 7.50, 1.50); 7.38–7.35 (1H, dd, *J* = 7.37, 1.30); 7.13–7.11 (1H, dd, *J* = 7.12, 1.10). δ_c_ (150 MHz, CDCl_3_): 181.95(C = O), 151.08, 146.95, 144.74, 144.01, 140.63, 132.81, 132.60, 131.58, 131.21, 130.77, 130.27, 129.52, 126.76, 126.41, 125.73, 125.41, 124.12, 113.81. UV-Visible λ_max_ (MeOH): 370.0 (3.81); 506.0 (3.24); 745.0 (3.87). HRMS (EI), *m/z* (% relative intensity): 71.0840 (2), 83.9540 (100), 301.0420 (2), 330.0464 [(100), M^+^]. Anal.calcd. for C_19_H_10_N_2_O_2_S: C, 69.08; H, 3.05; N, 8.48; S, 9.70. Found: C, 69.11; H, 3.07; N, 8.51; S, 9.74.

#### 6-(Furan-2-yl)-5*H*-naphtho[2,1-*b*]pyrido[2,3-*e*][1,4]oxazin-5-one (13) (Fig 3)

Procedure III was used to convert tributylfuranylstannane and 6-chloro-5*H*-naphtho[2,1-*b*]pyrido[2,3-*e*][1,4]oxazin-5-oneto the title product within 6 h. Analytically pure product obtained by flash chromatography employing 50% EtOAc/ 50% pet. ether eluent as a dark brown solid. Yield = 220 mg (70%). Mp. 210–212°C. NMR: δ_H_ (400 MHz, CDCl_3_): 8.84**–**8.82 (1H, m); 8.57–8.56 (1H, dd, *J* = 8.56, 1.56); 8.31–8.29 (1H, m); 7.77–7.69 (3H, m); 7.62–7.61(1H, dd, *J* = 7.61, 1.61); 7.40–7.34(2H, m); 6.59–6.58(1H, dd, *J* = 6.58, 1.58). δ_c_ (150 MHz, CDCl_3_): 180.98 (C = O), 151.89, 145.71, 145.66, 144.10, 143.26, 141.06, 133.07, 132.66, 131.88, 130.17, 126.62, 125.77, 125.70, 125.20, 116.44, 111.86, 110.72. UV-Visible λ_max_ (MeOH): 321.0 (4.05); 459.5 (3.87). Anal.calcd. for C_19_H_10_N_2_O_3_: C, 72.61; H, 3.21; N, 8.91. Found: C, 72.83; H, 3.17; N, 8.78.

### General Antimicrobial Sensitivity Testing of Compounds

A pure culture of human pathogenic microbes was obtained from culture collection center, Bishop Shahanan Hospital, Nsukka, Enugu State. The agar cup diffusion method was applied to determine the sensitivity of compounds against bacteria using Muller Hinton Agar. The MHA plates were inoculated with 1 x 10^4^ CFU culture of test organism. After which cups were made in each sector after previously dividing the plate into six segments and labeled. Using the sterile pipette, each cup was filled with four drops of compound (0.1 mg/ml). Pre-diffused time of 30 min was allowed before all the plates were incubated at 37°C for 24 h for bacteria. After incubation the inhibition zone diameter (IZD) resulting were measured and result recorded after subtracting the diameter of the cork borer. The cork borer used to make the cup is 8 mm in diameter. The procedure was repeated for tetracycline (standard bacteria) and DMSO (solvent).

### Minimum Inhibitory Concentration (MIC) Testing

The method used to determine the MIC was the same as for general sensitivity testing except serial dilution of 0.1 mg/ml DMSO solution of each sample was carried out to have 0.05, 0.025, 0.0125, 0.00625 mg/mL solutions. Fours drops of each dilution were added to the corresponding cup previously cut in the Mueller Hinton Agar (MHA) plate. The plates were incubated at 37°C for 24 h for bacteria and 48 h for fungi. The diameter of zone of inhibition was measured and the value subtracted from the diameter of the borer to give the inhibition zone diameter (IZD). The graph of IZD^2^ against the log of concentrations was plotted for each plate containing a specific compound and a microorganism. The anti-log of the intercept on x-axis gives the MIC. The procedure was repeated for tetracycline.

## Conclusion

The ease of accessing PBPs from periplasm and its absence in mammalian cells make them target of choice in search for antibiotics. The history of oxazines as having chemotherapeutic potential informed its usage as a parent molecule in the current study. The binding modes of two known oxazin-5-ones were used to guide the synthesis of derivatives. Evaluation of their SAR revealed that the analogues adopted a unique preferential configuration within the binding site cavity of the protein different from that of their parent molecules. This may account for the variation observed in their degree of PBP inhibition. Four of the analogues exhibited improved potencies over the parent molecules and were also drug-like according to Lipinski’s ro5. The biological assay results confirmed the antibiotic potencies of the derivatives, but were not in tandem with the computational predictions. Medicinal Chemists could take advantage of the ligand interaction motifs identified in this study in rational optimization by chemical modification of the compounds.

## Supporting Information

S1 Fig Predicted binding modes for all the derivatives (Part 4a), compounds with *K*_i_< 100 μM (Part b), derivatives with ethynyl and styryl phenyl substituents (Part c) and derivatives with hexynyl substituent (Part d). Polar contacts are shown as dashed lines. Carbons arecoloured green and gray, oxygens arecolouredred and nitrogens are coloured blue. Protein residues are represented in line format.
(TIF)
