# Supplementary figures and images for: Oxazin-5-Ones as a Novel Class of Penicillin Binding Protein Inhibitors: Design, Synthesis and Structure Activity Relationship
Source: PLoS One. 2016 Oct 17;11(10):e0163467. doi: 10.1371/journal.pone.0163467 (PMC5066960; doi:10.1371/journal.pone.0163467)

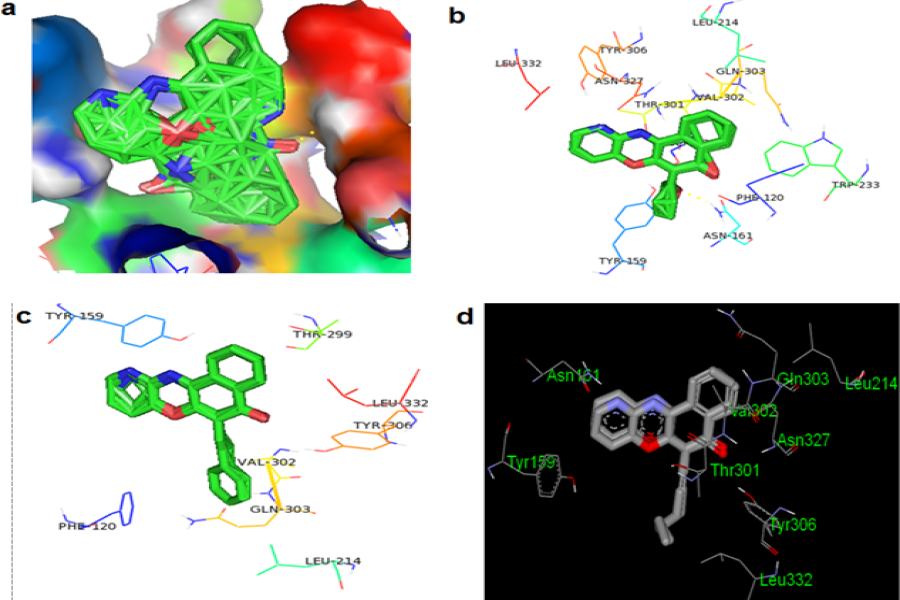

Supplement: S1 Fig — Predicted binding modes for all the derivatives (Part 4a), compounds with Ki< 100 μM (Part b), derivatives with ethynyl and styryl phenyl substituents (Part c) and derivatives with hexynyl substituent (Part d). Polar contacts are shown as dashed lines. Carbons arecoloured green and gray, oxygens arecolouredred and nitrogens are coloured blue. Protein residues are represented in line format. (TIF) [file pone.0163467.s001.tif]
